# Awareness and Potential Impacts of the Medicalization of Internet Gaming Disorder: Cross-sectional Survey Among Adolescents in China

**DOI:** 10.2196/22393

**Published:** 2021-02-24

**Authors:** Yanqiu Yu, Ji-Bin Li, Joseph T F Lau

**Affiliations:** 1 Center for Health Behaviours Research Jockey Club School of Public Health and Primary Care The Chinese University of Hong Kong Hong Kong Hong Kong; 2 Department of Clinical Research, Sun Yat-Sen University Cancer Center State Key Laboratory of Oncology in South China Collaborative Innovation Center for Cancer Medicine Guangzhou China

**Keywords:** gaming disorder, ICD-11, high-risk subgroups, disease awareness, medicalization, internet gaming, awareness, impact, adolescent, young adult, China, game, disorder, ICD

## Abstract

**Background:**

The Eleventh Revision of *International Classification of Diseases* (ICD-11) newly listed gaming disorder, including internet gaming disorder (IGD), as a disease. The level of awareness and potential positive and negative impacts of this medicalization among adolescents were unknown.

**Objective:**

This study investigated the levels, associated factors, and potential positive and negative impacts of awareness of the medicalization of IGD among adolescents in China.

**Methods:**

In a cross-sectional survey, 1343 middle school students in Guangzhou, China, self-administered an anonymous questionnaire in classrooms (October to December 2019). Three risk subgroups were identified: those who scored ≥5 items in the *Diagnostic and Statistical Manual of Mental Disorders, Fifth Edition* checklist (IGD-S), those who self-perceived having IGD currently (IGD-PC), and those who self-perceived having IGD within 12 months (IGD-P12M).

**Results:**

Of the internet gamers, 48.3% (460/952) were aware of the medicalization of IGD; they were more likely to belong to the IGD-P12M/IGD-S risk subgroups. Within the IGD-PC/IGD-P12M (but not IGD-S) risk subgroups, IGD medicalization awareness was positively associated with favorable outcomes (reduced internet gaming time in the past 12 months, seeking help from professionals if having IGD, and fewer maladaptive cognitions). After being briefed about the ICD-11 inclusion of IGD, 54.2% (516/952) and 32.8% (312/952) expressed that it would lead to the reduction of gaming time and help-seeking behaviors, respectively; however, 17.9% (170/952), 21.5% (205/952), 15.9% (151/952), and 14.5% (138/952) perceived self-doubt for being diseased, stronger pressure from family members, negative emotional responses, and labeling effect, respectively. With a few exceptions, such perceived positive or negative impacts were stronger among the IGD-S, IGD-PC, and IGD-P12M risk subgroups.

**Conclusions:**

The exploratory study shows that the medicalization of IGD may have benefits that need maximization and potentially harmful effects that need minimization. Future studies should test the efficacies of health promotion that increases IGD medicalization awareness.

## Introduction

Excessive internet gaming may cause a range of psychological and behavioral problems among adolescents [1,2]. Following the *Diagnostic and Statistical Manual of Mental Disorders, Fifth Edition* (DSM-5) definition announced in 2013, the World Health Organization (WHO) listed internet gaming disorder (IGD) as a subtype of gaming disorder in the *International Classification of Diseases, Eleventh Revision* (ICD-11) in 2018 [3] and formally endorsed the decision in May 2019 [4]. The classification of IGD as a disease reflects a medicalization process, which defines a health condition as a new disease that usually requires medical treatments [5]. Medicalization of diseases (eg, attention deficit hyperactivity disorder and social anxiety disorder) has been controversial [6-8]. The same is true for IGD. Supporters of the medicalization of IGD found similar neurological changes and addictive features among people with IGD and those with substance use disorders; they believed that medicalization would advance understandings of etiology, diagnosis, and treatment of IGD [9-18]. In contrast, the researchers who disagreed with this position were concerned about the absence of evidence-based treatment, overdiagnosis, and stigma toward heavily engaged internet gamers [19-21]. Medicalization’s potential benefits include new opportunities for treatments, awareness for prevention, and reduction of stigma by regarding affected people as patients instead people who are weak or have character flaws [5,9,10,17]. It is important to understand whether adolescents know about the medicalization of IGD and how they respond to it.

We contend that awareness of the official ICD-11 inclusion of IGD (represented by the term *IGD medicalization awareness* in this study) may increase positive coping behaviors that may lead to prevention (ie, reduction of gaming time in the past 12 months) and treatment (ie, intention to seek help from mental health professionals if having IGD) among adolescent internet gamers, especially those at higher risk of IGD. Such contentions have not been tested, but the belief that internet addiction is an illness was positively associated with willingness to change pathological internet habits [22]. Conceptually, IGD medicalization awareness may increase perceived severity of problematic internet gaming. Both the fear appeal theory [23] and the health belief model [24] postulate that perceived severity of a health-related problem is associated with the adoption of related preventive behaviors. In this study, at-risk adolescent internet gamers included those whose DSM-5 scores objectively exceeded the cutoff point (IGD-S) and those who subjectively perceived that they were having IGD currently (IGD-PC) or going to have IGD in the next 12 months (IGD-P12M). In the cases of internet addiction, which was significantly correlated with IGD [25], only 28.2% of those who self-perceived having internet addiction intended to correct their addiction problems [26]. Furthermore, those who were at high risk of internet addiction were even less likely than others to change their internet habits [22]. Improvements are needed to improve motivation to reduce unhealthy gaming behaviors among adolescents at risk of IGD; health promotion to increase their IGD medicalization awareness is potentially useful.

IGD medicalization awareness may alter maladaptive cognitions related to internet gaming, which are known determinants of IGD [27,28]. A study comprehensively reviewed such maladaptive cognitions and proposed a 4-factor structure that was used to construct the Internet Gaming Cognition Scale [27,29]. It was modified into a 3-factor scale (ie, the Chinese version of Revised Internet Gaming Cognition Scale), which was validated among Chinese adolescents [30] and used in this study. Those with IGD medicalization awareness might restructure their maladaptive cognitions. For instance, they might perceive internet gaming as less rewarding if they knew that it was a disease. We thus contended a negative association between IGD medicalization awareness and maladaptive cognitions related to internet gaming within the aforementioned risk subgroups of internet gamers.

Despite potential benefits, the medicalization of IGD may in parallel cause unintended negative consequences [19]. It may trigger unfavorable emotional responses among internet gamers, especially those at higher risk of IGD. According to the common sense model, illness representation that includes both cognitive and emotional representations may generate emotional responses to the disease of concern in both diseased people [31] and laypeople [32]. Hence, internet gamers with IGD medicalization awareness (especially those with self-perceived IGD) may generate negative emotions related to problematic internet gaming (eg, anxiety, guilt, blame, and shame). Second, the medicalization of IGD may induce stigma and self-stigma related to internet gaming [20,33]. Globally, people with mental illnesses encounter stigma [34]. Although the ICD-11 definition specifies that heavily engaged internet gamers who have not exhibited serious problems due to internet gaming in the past 12 months are not IGD cases [3], the general public may be unable to distinguish between heavily engaged (but healthy) gamers and IGD cases [20]. Third, parental control of adolescents’ internet gaming is common and often results in adolescent-parent conflicts [35]. When parents know about the medicalization of IGD, they may exert stronger pressure on adolescent internet gamers, enhancing their perceived stress [20]. Health workers hence need to alleviate potential negative consequences while pursuing the benefits of the medicalization of IGD. Perceptions of such positive and negative consequences of the medicalization of IGD have not been investigated.

Give such background, this study investigated (1) the prevalence of IGD medicalization awareness among adolescent internet gamers in mainland China; (2) adjusted associations between IGD medicalization awareness and reduction of internet gaming time (past 12 months), intention to seek help from mental health professionals if having IGD, and IGD-related maladaptive cognitions in 3 objectively and subjectively defined high-risk subgroups (IGD-S, IGD-PC, and IGD-P12M); (3) descriptions of perceived positive impacts (eg, reducing internet gaming time) and negative impacts (eg, labeling effect, emotional distress, and stronger pressure from family members) of the medicalization of IGD after participants were briefed about the inclusion of IGD into ICD-11 by the WHO; and (4) adjusted associations between the 3 types of IGD risk status and the aforementioned postbriefing perceived impacts.

## Methods

### Participants and Procedure

An anonymous cross-sectional survey was conducted among grade 8 (8 years of formal education) students of 4 secondary schools selected by nonrandom sampling from October to December 2019 in Guangzhou, China. Under the supervision of trained and experienced field workers, the students self-administered the questionnaire in the classroom setting without the presence of teachers. Participants were briefed that the return of the questionnaire implied informed consent. No incentives were given. The data collection procedure was described elsewhere [30]. Of the 1343 completed questionnaires (response rate of 99.1%), 1327 (98.8%) were valid. Data obtained from the 962 (72.5%) who had played internet games in the past 12 months were analyzed. The study was approved by the survey and behavioral research ethics committee of the Chinese University of Hong Kong (No. SBRE-18-430).

### Measures

#### Background Variables

Information about sex (male or female), living arrangement with parents (whether living with both parents, either of the parents, or neither of the parents), single-parent family status, relative household income to their classmates (much higher, higher, moderate, lower, or much lower), and self-reported academic performance (below average, average, or above average) was collected.

#### IGD Medicalization Awareness

The item was: “Do you know that IGD has been defined as a disease by the WHO (yes/no responses)?”

#### Objectively and Subjectively Defined IGD Risk Status

IGD-S was objectively defined as the endorsement of 5 or more of the 9 items of the validated Chinese version of the DSM-5 checklist [36,37]; Cronbach alpha was .74 in this study.

IGD-PC was assessed subjectively: “Do you think that you currently have IGD (yes=1, no=0)?”

IGD-P12M was subjectively assessed: “Do you think that you are going to have IGD in the next 12 months (yes=1, no=0)?” Similar questions on self-perceived IGD status have been used in previous internet addiction studies [26,38].

#### Maladaptive Cognitions Related to Internet Gaming

The validated 15-item Chinese version of the Revised Internet Gaming Cognition Scale has an overall scale and 3 subscales (0=never to 4=always) [30]. The overall scale was used in this report (Cronbach alpha .93).

#### Positive Coping Behavior/Intention

The two items, answered yes=1 or no=0, were “Have you reduced internet gaming time in the past 12 months?” and “Would you seek help from mental health professionals if you have IGD?”

#### Postbriefing Perceived Impacts of the Medicalization of IGD

After being briefed that “The WHO approved the ICD-11 on May 25, 2019, which defined IGD as a disease. The member states of the WHO should develop their new treatment and prevention policies prior to January l, 2022,” participants rated a 6-item checklist (yes/no responses) on whether the new ICD-11 definition of IGD (medicalization) would impact them positively (ie, leading to participants’ reduction of gaming time and seeking help from others) or negatively (ie, the news would lead to self-doubt being diseased, increase in parental pressure against playing internet games, labeling effect, and emotional distress due to playing internet games). These questions were asked at the last part of the questionnaire and thus could not affect the responses to the other questions.

### Statistical Analysis

Logistic regression analyses were performed to investigate the associations involving binary outcomes, adjusted for background variables. Adjusted odds ratios and 95% confidence intervals were reported. Analysis of covariance was performed to compare between-group differences in the continuous dependent variables, adjusted for background variables. Cohen *d* represented the effect sizes of the between-group differences. SPSS Statistics 21.0 (IBM Corporation) was used for data analysis; 2-tailed *P*<.05 and .05<*P*<.10 denoted statistical significance and marginal statistical significance, respectively.

## Results

### Descriptive Statistics

The results are presented in Table 1. About two-thirds (601/952, 63.1%) of the internet gamers were males; 14.0% (133/952) did not live with both parents; 11% (105/952) came from single-parent families; 9.6% (91/952) perceived lower/much lower household income relative to classmates; 27.3% (260/952) self-reported below-average academic performance. Of the internet gamers, 10.8% (103/952), 58.9% (561/952), and 60.5% (576/952) belonged to the IGD-S, IGD-PC, and IGD-P12M risk subgroups, respectively (see Table 1). Within such 3 subgroups, 50.5% (52/103), 62.6% (351/561), and 65.5% (377/576) self-reported that they had reduced internet gaming time in the past 12 months (69.1% [658/952] among all gamers), and 31.1% (32/103), 39.4% (221/561), and 43.6% (251/576) reported that they would seek help from mental health professionals if having IGD (44.9% [427/952] among all gamers), respectively.

**Table 1:** Descriptive statistics of the participants (n=952).

Characteristics			Value, n (%)
**Background variables**			
	**Sex**		
		Female	351 (36.9)
		Male	601 (63.1)
	**Living arrangement with both parents**		
		Yes	818 (85.9)
		No	133 (14.0)
		Missing data	1 (0.1)
	**Single-parent family status**		
		No	844 (88.7)
		Yes	105 (11.0)
		Missing data	3 (0.3)
	**Household income relative to classmates**		
		Higher/much higher	282 (29.6)
		Moderate	570 (59.9)
		Lower/much lower	91 (9.6)
		Missing data	9 (0.9)
	**Self-reported academic performance**		
		Above average	198 (20.8)
		Average	492 (51.7)
		Below average	260 (27.3)
		Missing data	2 (0.2)
**IGD^a^ status (scored or perceived)**			
	**DSM-5^b^ scored IGD**		
		No	845 (88.8)
		Yes	103 (10.8)
		Missing data	4 (0.4)
	**Self-perceived having IGD currently**		
		No	385 (40.4)
		Yes	561 (58.9)
		Missing data	6 (0.6)
	**Going to have IGD in the next 12 months**		
		No	364 (38.2)
		Yes	576 (60.5)
		Missing data	12 (1.3)
	**Any of the above (scored or perceived IGD)**		
		No	259 (27.2)
		Yes	687 (72.2)
		Missing data	6 (0.6)
**IGD medicalization awareness**			
	No		471 (49.5)
	Yes		460 (48.3)
	Missing data		21 (2.2)

^a^IGD: internet gaming disorder.

^b^DSM-5: *Diagnostic and Statistical Manual of Mental Disorders, Fifth Edition.*

### Prevalence and Factors of IGD Medicalization Awareness

Of the internet gamers, 48.3% (460/952) reported IGD medicalization awareness (see Table 1). No background variables were significantly associated with IGD medicalization awareness (see Table 2). Adjusted for all the studied background variables, the negative association between IGD-P12M status and IGD medicalization awareness (adjusted odds ratio [AOR] 0.76, 95% CI 0.58-0.99) was statistically significant; the negative association between IGD-S status and IGD medicalization awareness was of marginal statistical significance (AOR 0.65, 95% CI 0.42-1.01; *P*=.056); that between IGD-PC status and IGD medicalization awareness was statistically nonsignificant (see Table 2).

**Table 2:** Factors of internet gaming disorder medicalization awareness^a^ (n=952).

Characteristic			IGD^b^ medicalization awareness		
			n (%)	ORu^c^ (95% CI)	AOR^d^ (95% CI)
**Background variables**					
	**Sex**				
		Female	176 (50.6)	1	—
		Male	284 (48.7)	0.93 (0.71-1.21)	—
	**Living arrangement with both parents**				
		Yes	404 (50.3)	1	—
		No	55 (43.3)	0.75 (0.52-1.10)	—
	**Single-parent family status**				
		No	413 (50.0)	1	—
		Yes	45 (44.1)	0.79 (0.52-1.19)	—
	**Household income relative to classmates**				
		Higher/much higher	145 (52.7)	1	—
		Moderate	268 (47.9)	0.83 (0.62-1.10)	—
		Lower/much lower	42 (47.2)	0.80 (0.50-1.29)	—
	**Self-reported academic performance**				
		Above average	101 (52.1)	1	—
		average	237 (49.1)	0.89 (0.64-1.24)	—
		Below average	121 (48.0)	0.85 (0.59-1.24)	—
**IGD status**					
	**DSM-5^e^ scored IGD**				
		No	417 (50.4)	1	1
		Yes	40 (40.0)	0.66 (0.43-1.00)	0.65 (0.42-1.01)
	**Self-perceived having IGD currently**				
		No	203 (53.1)	1	
		Yes	255 (46.6)	0.77 (0.59-1.00)	0.80 (0.61-1.05)
	**Going to have IGD in the next 12 months**				
		No	196 (54.4)	1	1
		Yes	262 (46.5)	0.73 (0.56-0.95)	0.76 (0.58-0.99)
	**Any of the above (scored or perceived IGD)**				
		No	144 (56.3)	1	1
		Yes	314 (46.7)	0.68 (0.51-0.91)	0.71 (0.53-0.96)

^a^Missing data were excluded from the analyses.

^b^IGD: internet gaming disorder.

^c^ORu: univariate odds ratio.

^d^AOR: adjusted odds ratio.

^e^DSM-5: *Diagnostic and Statistical Manual of Mental Disorders, Fifth Edition*.

### Associations Between IGD Medicalization Awareness and IGD and Potential Outcomes Within the Three High-Risk Subgroups

**Figure 1:**
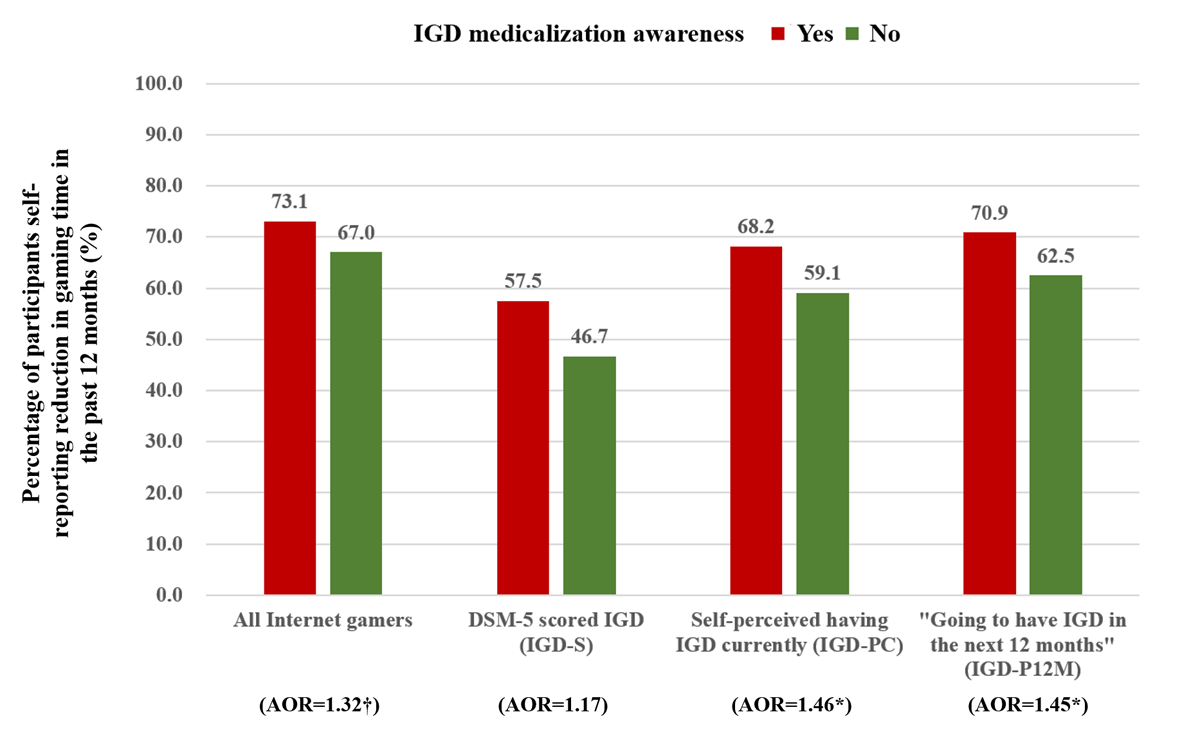
Comparing percentages of participants self-reporting reduction in gaming time between those with and without internet gaming disorder medicalization awareness. IGD: internet gaming disorder; DSM-5: DSM-5: Diagnostic and Statistical Manual of Mental Disorders, Fifth Edition; AOR: adjusted odds ratio. (†: .05<*P*<.10; *: *P*<.05).

**Figure 2:**
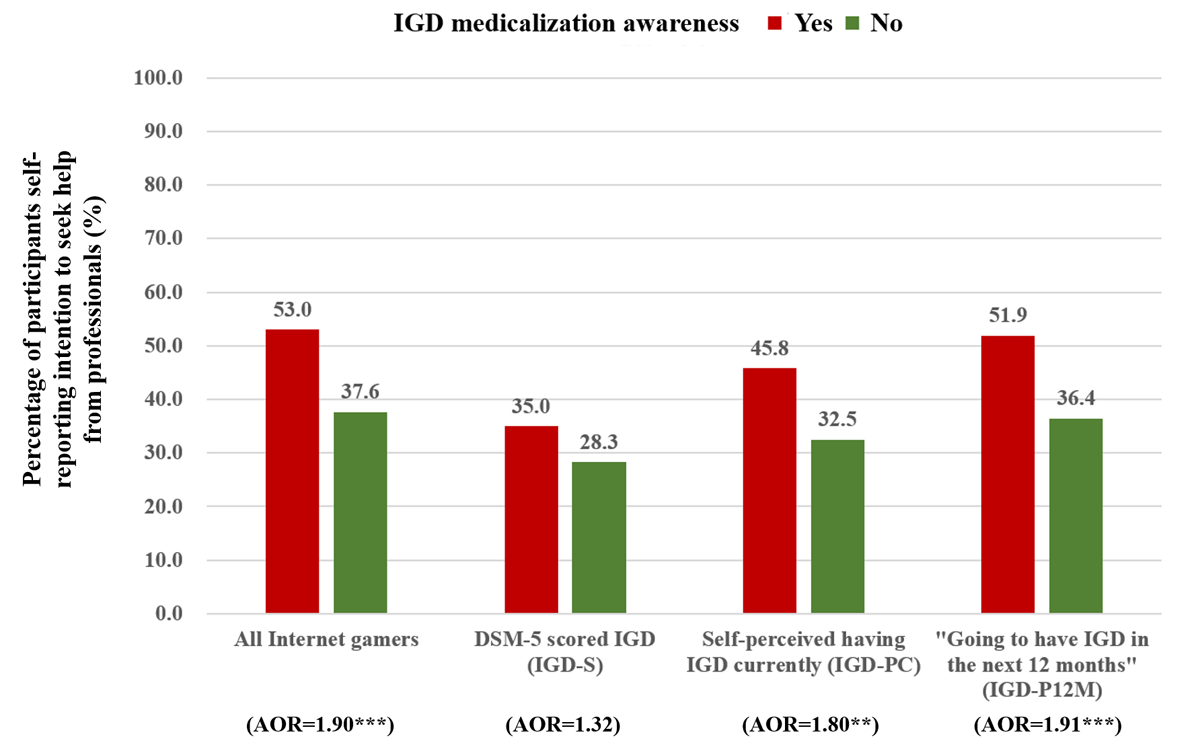
Comparing percentages of participants self-reporting intention to seek help from professionals between those with and without internet gaming disorder medicalization awareness. IGD: internet gaming disorder; DSM-5: DSM-5: Diagnostic and Statistical Manual of Mental Disorders, Fifth Edition; AOR: adjusted odds ratio. (**: *P*<.01; ***: *P*<.001).

In the IGD-PC and IGD-P12M subgroups, IGD medicalization awareness was significantly associated with the reduction in gaming time in the past 12 months (AOR 1.46 and AOR 1.45, respectively) and the intention to seek professional help if having IGD (AOR 1.80 and AOR 1.91, respectively). Such associations were, however, not statistically significant in the IGD-S subgroup. Among all internet gamers, IGD medicalization awareness was significantly associated with the intention to seek help from mental health professionals if having IGD (AOR 1.90, 95% CI 1.45-2.47); the association between IGD medicalization awareness and reduction in internet gaming time in the past 12 months was of marginal statistical significance (AOR 1.32, 95% CI 0.99-1.76; *P*=.06; see Figures 1 and 2). The adjusted analysis of covariance in Table 3 showed a similar pattern. The association between IGD medicalization awareness and maladaptive cognitions was not significant in the IGD-S subgroup but was statistically significant in the IGD-P12M subgroup (Cohen *d*=0.24, *P*=.01), marginally significant in the IGD-PC subgroup (Cohen *d=*0.18; *P*=.07), and significant among all internet gamers (Cohen *d*=0.18, *P*=.02).

**Table 3:** Associations between internet gaming disorder medicalization awareness and preventive behavior/intention in the three high-risk subgroups^a^.

Overall maladaptive cognitions	IGD^b^ medicalization awareness			
	Yes, mean (SD)	No, mean (SD)	*P* value	Cohen *d*
DSM-5^c^ scored IGD (n=100)	32.5 (14.1)	33.2 (12.0)	.82	.09
Self-perceived having IGD currently (n=582)	23.6 (11.2)	25.5 (11.4)	.07	.18
Going to have IGD in the next 12 months (n=576)	22.3 (11.0)	25.0 (11.5)	.01	.24
All internet gamers (n=952)	20.2 (11.1)	22.2 (11.8)	.02	.18

^a^Missing data were excluded from the analyses.

^b^IGD: internet gaming disorder.

^e^DSM-5: *Diagnostic and Statistical Manual of Mental Disorders, Fifth Edition.*

### Perceived Impacts of the Medicalization of IGD and Associations With Risk Status of IGD

After being briefed about the new inclusion of IGD into the ICD-11 by the WHO (see Measurements), 54.2% (516/952) of all the internet gamers indicated that this knowledge would make them spend less time on internet gaming, while 32.8% (312/952) indicated that it would drive them to seek help from others to deal with problems related to internet gaming (see Table 4). Besides, 17.9% (170/952), 21.5% (205/952), 15.9% (151/952), and 14.5% (138/952) of the internet gamers, after being briefed about the medicalization, perceived that it would subject them to self-doubt for being diseased, stronger pressure from family members, development of negative emotions (eg, anxiety), and label as being sick, respectively (see Table 4).

IGD-PC and IGD-P12M status but not IGD-S status were positively associated with the two perceived positive impacts (reduction in internet gaming time and intention to seek help from others to deal with problems related to internet gaming) at significant or marginally significant levels. Moreover, IGD-S, IGD-PC, and IGD-P12M status were all positively and significantly associated with the 4 types of perceived negative impacts (AOR ranged from 1.69 to 3.23) except for one association (that between IGD-P12M status and labeling effect) of marginal significance (AOR 1.50, 95% CI 1.00-2.26; *P*=.05; see Table 4).

**Table 4:** Perceived impacts of the International Classification of Diseases, Eleventh Revision inclusion of internet gaming disorder among internet gamers^a^ (n=952).

Outcomes		IV^b^=yes, n (%)	IV=no, n (%)	AOR^c^ (95% CI)
**DSM-5^d^ scored IGD^e^**				
	Intend to reduce gaming time	49 (47.6)	465 (55.0)	0.78 (0.51-1.20)
	Intend to seek help from others	34 (33.0)	277 (32.8)	0.99 (0.63-1.57)
	Self-doubt for being diseased	33 (32.0)	136 (16.1)	2.63 (1.62-4.26)
	Stronger pressure from family members	46 (44.7)	159 (18.8)	3.23 (2.07-5.04)
	Being labeled as being sick	27 (26.2)	111 (13.1)	2.23 (1.35-3.70)
	Negative emotions	33 (32.0)	117 (13.8)	2.96 (1.83-4.79)
**Self-perceived having IGD currently**				
	Intend to reduce gaming time	321 (57.2)	193 (50.1)	1.34 (1.02-1.76)
	Intend to seek help from others	196 (34.9)	115 (29.9)	1.31 (0.98-1.76)
	Self-doubt for being diseased	125 (22.3)	45 (11.7)	1.92 (1.31-2.83)
	Stronger pressure from family members	149 (26.6)	54 (14.0)	2.01 (1.41-2.87)
	Being labeled as being sick	101 (18.0)	36 (9.4)	1.94 (1.27-2.96)
	Negative emotions	106 (18.9)	45 (11.7)	1.69 (1.14-2.50)
**Going to have IGD in the next 12 months**				
	Intend to reduce gaming time	327 (56.8)	184 (50.5)	1.28 (0.98-1.68)
	Intend to seek help from others	201 (34.9)	108 (29.7)	1.31 (0.98-1.75)
	Self-doubt for being diseased	128 (22.2)	41 (11.3)	2.10 (1.42-3.12)
	Stronger pressure from family members	157 (27.3)	45 (12.4)	2.61 (1.80-3.79)
	Being labeled as being sick	95 (16.5)	40 (11.0)	1.50 (1.00-2.26)
	Negative emotions	118 (20.5)	31 (8.5)	2.81 (1.82-4.33)

^a^Missing data were excluded from the analyses.

^b^IV: independent variable.

^c^AOR: adjusted odds ratio.

^d^DSM-5: *Diagnostic and Statistical Manual of Mental Disorders, Fifth Edition*.

^e^IGD: internet gaming disorder.

Adjusted logistic regression models used those whose IGD risk status endorsing no as the reference groups (versus yes) and adjusted for background factors, including sex, living arrangement with both parents, single-parent family status, relative household income to their classmates, and self-reported academic performance.

## Discussion

### Principal Findings

In general, ICD-11 is highly influential [39]. The ICD-11 inclusion of IGD requires all nations to establish related prevention and treatment policies [9-11,13,14,16-18]. It is hence an expected driving force to reduce IGD worldwide. Health workers need to increase its benefits and reduce unintended negative consequences. It is essential to disseminate information about the new ICD-11 inclusion of IGD to adolescents and stakeholders (eg, parents, teachers, health workers, and social workers) as our data showed that the IGD medicalization awareness may reduce adolescent risky gaming behaviors and maladaptive cognitions related to internet gaming. It is equally important to understand adolescents’ cognitive, behavioral, and emotional responses to the medicalization of IGD. This study filled out such knowledge gaps. There was no apparent social disparity in the IGD medicalization awareness as it was not associated with the studied background variables. Nonetheless, IGD medicalization awareness was lower in 2 high-risk subgroups (IGD-S [.05<*P*<.10] and IGD-P12M [*P*<.05]); the promotion of the disease awareness should thus target at-risk adolescents.

It is encouraging that adolescents possessing IGD medicalization awareness were more likely than their counterparts to have (1) reduced gaming time in the last 12 months, (2) intention to seek help from professionals if having IGD, and (3) fewer IGD-related maladaptive cognitions. It is plausible that the knowledge about the medicalization of IGD may have enhanced adolescents’ perceived severity of playing internet games excessively and motivations to take up preventive measures (eg, reducing gaming time and seeking help) according to the fear appeal theory [23] and the health belief model [24]. Such observed associations were triangulated by the encouraging finding that, similarly, many internet gamers indicated that they would reduce gaming time and seek help from others after being briefed about the medicalization of IGD. In the future, randomized controlled trials (RCTs) should be conducted to compare the efficacies of interventions providing adolescents IGD-related health promotion materials with and without additional information on the medicalization of IGD in fostering positive outcomes in terms of perceptions, mental distress, and behaviors related to IGD.

The associations between IGD medicalization awareness and the potential positive coping behavior/intention were more likely to be statistically significant within the 2 subjectively defined risk groups (IGD-PC and IGD-P12M) than within the objectively defined IGD group (IGD-S). The conceptual difference between diseases and illnesses is noteworthy. Diseases refer to objective clinical diagnoses, while illnesses refer to subjective experiences related to mental or physical symptoms [40,41]. The IGD-S subgroup was identified by the DSM-5 using a biomedical disease model, while the IGD-PC and IGD-P12M subgroups were subjectively evaluated and closer to the illness model. Understandably, those with illness perceptions (subjective beliefs of oneself being ill or going to be ill) were more prone to adopt positive corrective coping behaviors than those being objectively defined as IGD cases who might not feel ill. Besides, according to the health belief model [24], subjective perceptions of illness may be seen as a cue to action, which is a determinant of health-related behaviors (positive coping behaviors in our case).

Importantly, about one-fifth of the adolescent internet gamers showed concerns about side effects of the medicalization of IGD (eg, self-doubt about being diseased and worry about labeling effect). According to the common sense model [31], such problems may lead to mental health problems (eg, depression). Understandably, our data showed that the 3 at-risk subgroups were more likely than others to perceive the aforementioned negative consequences of the medicalization of IGD. To reduce such stigma, health education needs to clarify the distinction between heavily engaged internet gamers and disordered gamers.

### Limitations

Although this study is possibly the first one to investigate awareness of the ICD-11 inclusion of IGD, it has some limitations. First, the findings of this study are exploratory in nature and need to be confirmed by longitudinal studies and RCTs. Second, the cross-sectional study design did not allow for the establishment of causality. Third, generalization of the results should be done with caution, as a limited number of schools were selected nonrandomly in one city in mainland China. Fourth, IGD medicalization awareness and potential responses to the medicalization (eg, reduction in gaming time) were assessed by self-reported single items that have not been validated. Fifth, social desirability bias might have inflated the levels of IGD medicalization awareness and positive coping behavior/intention. Sixth, the immediate postbriefing responses may not be reliable and may differ from actual behaviors.

### Conclusions

Less than half the adolescent participants knew about the medicalization of IGD indicating there is room for improvement. The associations between IGD medicalization awareness and favorable coping behavior/intention/cognitions are encouraging. Dissemination of information about the inclusion of IGD into ICD-11 may induce adolescents to take up preventive and/or help-seeking behaviors. Such may be especially true within high-risk subgroups. Future RCTs are thus warranted to support the development of a simple, sustainable, and well-documented intervention that can be used to increase disease awareness of IGD among adolescents, possibly incorporating health promotion of healthy internet gaming. Through implementation research, such an evidence-based intervention can further be scaled up and used across countries. Furthermore, health workers need to minimize potential negative impacts of the medicalization (eg, avoidance of overpathologizing internet gamers). Research should also look at IGD medicalization awareness among other stakeholders (eg, parents, teachers, and social workers). This exploratory study is a starting point to understand the importance of potential effects of the medicalization of IGD.

## Abbreviations

AOR: adjusted odds ratio
DSM-5: *Diagnostic and Statistical Manual of Mental Disorders, Fifth Edition*
ICD-11: *International Classification of Diseases, Eleventh Revision*
IGD: internet gaming disorder
IGD-S: Scored IGD status
IGD-P12M: Perceived future IGD status in the next 12 months
IGD-PC: Perceived current IGD status
RCT: randomized controlled trial
WHO: World Health Organization
